# Alterations in hippocampal serotonergic and INSR function in streptozotocin induced diabetic rats exposed to stress: neuroprotective role of pyridoxine and *Aegle marmelose*

**DOI:** 10.1186/1423-0127-17-78

**Published:** 2010-09-25

**Authors:** Pretty Mary Abraham, Korah P Kuruvilla, Jobin Mathew, Anitha Malat, Shilpa Joy, CS Paulose

**Affiliations:** 1 Molecular Neurobiology and Cell Biology Unit, Centre for Neuroscience, Department of Biotechnology, Cochin University of Science and Technology, Cochin- 682 022, Kerala, India

## Abstract

Diabetes and stress stimulate hippocampal 5-HT synthesis, metabolism and release. The present study was carried out to find the effects of insulin, *Aegle marmelose *alone and in combination with pyridoxine on the hippocampal 5-HT, 5-HT_2A _receptor subtype, gene expression studies on 5-HT_2A_, 5-HTT, INSR, immunohistochemical studies and elevated plus maze in streptozotocin induced diabetic rats. 5-HT content showed a significant decrease (*p *< 0.001) and a significant increase (*p *< 0.001) in 5-HIAA in hippocampus of diabetic rats compared to control. 5-HT receptor binding parameters B_max _and K_d _showed a significant decrease (*p *< 0.001) whereas 5-HT_2A _receptor binding parameters B_max _showed a significant decrease (*p *< 0.001) with a significant increase (*p *< 0.05) in K_d _in hippocampus of diabetic rats compared to control. Gene expression studies of 5-HT_2A, _5-HTT and INSR in hippocampus showed a significant down regulation (*p *< 0.001) in diabetic rats compared to control. Pyridoxine treated in combination with insulin and *A. marmelose *to diabetic rats reversed the 5-HT content, B_max _, K_d _of 5-HT, 5-HT_2A _and gene expression of 5-HT_2A_, 5-HTT and INSR in hippocampus to near control. The gene expression of 5-HT_2A _and 5-HTT were confirmed by immunohistochemical studies. Behavioural studies using elevated plus maze showed that serotonin through its transporter significantly increased (p < 0.001) anxiety-related traits in diabetic rats which were corrected by combination therapy. Our results suggest that pyridoxine treated in combination with insulin and *A. marmelose *has a role in the regulation of insulin synthesis and release, normalising diabetic related stress and anxiety through hippocampal serotonergic function. This has clinical significance in the management of diabetes.

## Background

Diabetes is associated with several adverse effects on the brain, which results primarily from direct consequences of chronic hyperglycemia. Diabetes induces impairments in hippocampal synaptic plasticity, neurogenesis and associated cognitive deficits. Intrahippocampal insulin [1] or activation of insulin signalling pathways [2] block the effects of stress on learning and memory. In control rats, hippocampus dependent learning is correlated with a decrease in extracellular glucose, and intrahippocampal injection of glucose improves performance [3]. Learning-induced changes in hippocampal glucose metabolism have been demonstrated in diabetic rats [4]. Hippocampus is particularly susceptible to the negative consequences of diabetes [5]. Individuals with diabetes suffer from reduced motor activity and are at increased risk of dementia and cognitive dysfunction [6]. 5-HT innervations of the hippocampus originate from the raphe nuclei in the midbrain [7]. 5-HT is released into the extracellular space and *via *synapses [8]. Direct effects of 5-HT on principal cells occur through its release in extracellular space. 5-HT_2A _receptors are involved in a diversity of physiological functions such as the control of nociception, motor behaviour, endocrine secretion, thermoregulation and modulation of appetite [9].

There is a need to explore diabetes and its complications to reduce the mechanisms by which oxidative stress develop diabetic complications. In an effort to expand the treatment, *Aegle marmelose *(L.) Correa ex Roxb. an ayurvedic medicinal tree, growing throughout the deciduous forest of India is reported to have anti-diabetic effect in rats. In the brain, L-tryptophan is converted to 5-HT in the presence of the co-enzyme pyridoxine [10]. 5-HT decrease has been reported in hypothyroidism and hypertension [9,11]. Pyridoxine supplementation is used for cognitive impairment or dementia [12].

In the current study, the effect of leaf extract of *Aegle marmelose *and insulin alone and in combination with pyridoxine in diabetic rats on the hippocampal 5-HT through 5HT_2A _receptor subtype - 5HT_2A_, 5-HTT and INSR gene expression and immunohistochemical studies using confocal microscope was carried out. Behavioural studies using elevated plus maze was also done to elucidate the anxiety-related traits in these rats.

## Materials and methods

### Animals

Adult Male Wistar rats 200 - 250 g body weight were purchased from Amrita Institute of Medical Sciences, Cochin and used for all experiments. They were housed in separate cages under 12 hours light and 12 hours dark periods and were maintained on standard food pellets, water *ad libitum *and room temperature. They were housed for 1 to 2 weeks before experiments were performed. All animal care and procedures were in accordance with Institutional and National Institute of Health guidelines.

### Induction of Diabetes

The animals were randomly divided into control (C), diabetic (D), insulin treated diabetic (D+I), diabetic treated with insulin + pyridoxine (DIP), diabetic treated with pyridoxine alone (D+P), diabetic treated with *Aegle marmelose *(D+A) and diabetic treated with *Aegle marmelose *+ pyridoxine (DAP). Each group consisted of 6-8 animals. Values are mean ± S.E.M of 4-6 rats in each group. Diabetes was induced by a single intrafemoral dose (55 mg/kg body weight) of streptozotocin prepared in citrate buffer, pH 4.5 [13]. The D+I and DIP groups received a daily dose (1 Unit/kg body weight) of Lente and Plain insulin. Pyridoxine injected was 100 mg/kg body weight [14]. Aqueous extract of *Aegle marmelose *was given orally in the dosage of 1 g/Kg body weight [15] at 24 hour intervals. The experimental rats were sacrificed by decapitation after 15 days treatment. The hippocampus was dissected out quickly over ice according to the procedure of [16]. The tissues were stored at -80°C until assay. Glucose was measured by GOD-POD glucose estimation kit (Biolab Diagnostics Pvt. Ltd).

### Plant material and Preparation of extract

Specimen of *Aegle marmelose *were collected and voucher specimens was deposited at herbarium of Centre for Neuroscience, Cochin University of Science and Technology, Cochin, Kerala, India. Fresh leaves of *Aegle marmelose *were air dried in shade and powdered. 10 g of leaf powder was mixed with 100 ml of distilled water and stirred for 2 hr. It was kept overnight at 4°C. The supernatant was collected and evaporated to dryness followed by lyophylization in Yamato, Neocool, Japan lyophilizer. This was used as the crude leaf extract to study the antidiabetic effect in streptozotocin induced diabetes.

### Quantification of Serotonin

Serotonin content was assayed according to Paulose et al. [17]. The cerebral cortex and brain stem of the experimental groups of rats was homogenized in 0.4 N perchloric acid. The homogenate was centrifuged at 5000 × g for 10 min at 4°C in a Sigma 3K30 refrigerated centrifuge and the clear supernatant was filtered through 0.22 μm HPLC grade filters and used for HPLC analysis.

Serotonin (5-HT) and 5-hydroxy indole acetic acid (5-HIAA) contents were determined using high performance liquid chromatography integrated with an electrochemical detector (HPLC-ECD) (Waters, USA) fitted with CLC-ODS reverse phase column of 5 μm particle size. The mobile phase consisted of 50 mM sodium phosphate dibasic, 0.03 M citric acid, 0.6 mM sodium octyl sulphonate, 0.1 mM EDTA and 15% methanol. The pH was adjusted to 3.25 with orthophosphoric acid, filtered through the 0.22 μm filter (Millipore) and degassed. A Waters model 515, Milford, USA, pump was used to deliver the solvent at a rate of 1 ml/minute. The neurotransmitters and their metabolites were identified by amperometric detection using an electrochemical detector (Waters, model 2465) with a reduction potential of +0.80 V.

### 5-HT Receptor Binding Studies Using [^3^H] 5-Hydroxytryptamine

5-HT receptor assay was done using [^3^H] 5-hydroxytryptamine binding in crude synaptic membrane preparations of hippocampus by the modified method of [18]. Crude membrane preparation was suspended in 50 mM Tris-HCl buffer, pH 8.5, containing 1.0 μM paragyline. The incubation mixture contained 0.3-0.4 mg protein. In the saturation binding experiments, assays were done using different concentrations i.e., 1.0 nM-30 nM of [^3^H] 5-HT was incubated with and without excess of unlabelled 10 μM 5-HT. Tubes were incubated at 37°C for 15 min. and filtered rapidly through GF/B filters (Whatman). The filters were washed quickly by three successive washing with 5.0 ml of ice cold 50 mM Tris buffer, pH 8.5. Bound radioactivity was counted with cocktail-T in a Wallac 1409 liquid scintillation counter.

### 5-HT_2A _Receptor Binding Studies Using [^3^H] Ketanserin

5-HT_2A _receptor assay was done using [^3^H] Ketanserin binding in crude synaptic membrane preparations of hippocampus by the modified method of [19]. Crude membrane preparation was suspended in 50 mM Tris-HCl buffer, pH 7.6. The incubation mixture contained 0.3-0.4 mg protein. In the saturation binding experiments using different concentrations i.e., 0.1 nM - 2.5 nM of [^3^H] Ketanserin was incubated with and without excess of unlabelled 10 μM Ketanserin. Tubes were incubated at 37°C for 15 minutes and filtered rapidly through GF/B filters (Whatman). The filters were washed quickly by three successive washing with 5.0 ml of ice cold 50 mM Tris buffer, pH 7.6. Bound radioactivity was counted with cocktail-T in a Wallac 1409 liquid scintillation counter. Protein was measured by the method of Lowry et al. [20] using bovine serum albumin as standard.

### Receptor data analysis

The data were analysed according to Scatchard [21]. The binding parameters, maximal binding (B_max_) and equilibrium dissociation constant (K_d_), were derived by linear regression analysis.

### Real -Time PCR Assay using 5-HT_2A_, 5-HTT and INSR

RNA was isolated from the hippocampus of experimental rats using the Tri reagent (MRC, USA). Total cDNA synthesis was performed using ABI PRISM cDNA archive kit in 0.2 ml microfuge tubes. The reaction mixture of 20 μl contained 0.2 μg total RNA, 10 × RT buffer, 25 × dNTP mixture, 10 × random primers, MultiScribe RT (50 U/μl) and RNase free water. The cDNA synthesis reactions were carried out at 25°C for 10 minutes and 37°C for 2 hours using an Eppendorf Personal Cycler. Total cDNA synthesis was performed using ABI PRISM cDNA Archive kit. Real-Time PCR assays were performed in 96-well plates in ABI 7300 Real-Time PCR instrument (Applied Biosystems). PCR analyses were conducted with gene-specific primers and fluorescently labelled Taqman 5-HT receptor subtype (5HT_2A_; Rn01468302_m1), 5-HT transporter (5HTT; Rn00564737_m1) and Insulin receptor (INSR; Rn00567070)) (designed by Applied Biosystems). Endogenous control (β-actin) was labelled with a report dye (VIC). The real-time data were analyzed with Sequence Detection Systems software version 1.7. All reactions were performed in duplicate.

The ΔΔCT method of relative quantification was used to determine the fold change in expression. This was done by first normalizing the resulting threshold cycle (CT) values of the target mRNAs to the CT values of the internal control β-actin in the same samples (ΔCT = CT _Target _- CT _β-actin_). It was further normalized with the control (ΔΔCT = ΔCT - CT _Control_). The fold change in expression was then obtained (2^-ΔΔCT^).

### 5-HT_2A _and 5-HTT Expression Studies in the Hippocampus of control and experimental rats using confocal microscope

Control and experimental rats were anesthetized with ether. The rat was transcardially perfused with PBS, pH 7.4, followed by 4% paraformaldehyde in PBS [22]. After perfusion the brains were dissected and immersion fixed in 4% paraformaldehyde for 1 hr and then equilibrated with 30% sucrose solution in 0.1 M PBS, pH 7.0. 40 μm sections were cut using Cryostat (Leica, CM1510 S). The sections were treated with PBST (PBS in 0.01% Triton X-100) for 20 min. Brain slices were incubated overnight at 4°C with either rat primary antibody for 5-HT_2A _(No: RA24288 BD PharmenginTM, diluted in PBST at 1: 500 dilution) and 5HTT (No: AB9726 Chemicon Temecula, diluted in PBST at 1: 500 dilution). After overnight incubation, the brain slices were rinsed with PBST and then incubated with appropriate secondary antibody of either FITC (No: AB7130F, Chemicon, diluted in PBST at 1: 1000). The sections were observed and photographed using confocal imaging system (Leica SP 5).

### Elevated plus maze

The elevated plus-maze is a widely used animal model of anxiety that is based on two conflicting tendencies; the rodents drive to explore a novel environment and its aversion to heights and open spaces. Four arms were arranged in the shape of a cross. Two arms had side walls and an end wall ("closed arms") - the two other arms had no walls ("open arms"). The open arms were surrounded by small ledges to prevent the animal from falling from the maze. The maze was fastened to a light-weight support frame. Thus "anxious" animals spent most of the time in the closed arms while less anxious animals explored open areas longer.

#### Procedure

Animals were placed individually into the center of elevated plus-maze consisting of two open arms (38 L × 5 W cm) and two closed arms (38 L × 5 W × 15 H cm), with a central intersection (5 cm × 5 cm) elevated 50 cm above the floor. Behaviour was tested in a dimly lit room with a 40 W bulb hung 60 cm above the central part of the maze. The investigator sitting approximately 2 m apart from the apparatus observed and detected the movements of the rats for a total of 5 minutes. The experimental procedure was similar to that described by [23]. During the 5 min test period the following parameters were measured to analyze the behavioural changes of the experimental rats using elevated plus-maze: open arm entry, closed arm entry, percentage arm entry, total arm entry, time spent in open arm, time spent in closed arm, percentage of time spent in open arm [24,25]. An entry was defined as entering with all four feet into one arm. A decrease in open arm entries and decrease in time spent in the open arms is indicative of anxiogenic activity shown by experimental rats.

### Statistical Analysis

The equality of all the groups was tested by the analysis of variance (ANOVA) technique for different values of p. Further the pair wise comparisons of all the experimental groups were studied using Students-Newman-Keuls test at different significance levels. The testing was performed using GraphPad Instat (Ver. 2.04a, San Diego, USA) computer program.

## Results

### Estimation of blood glucose

**Figure 1:**
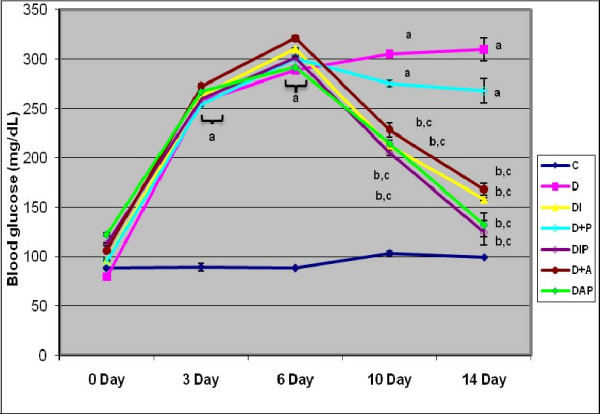
**Representative graph showing Blood glucose (mg/dl) level in Control and Experimental rats**. Values are mean ± S.E.M of 4-6 rats in each group. Each group consists of 6-8 rats. ^a ^p < 0.001 when compared to control; ^b ^p < 0.001 when compared to diabetic group; ^c ^p < 0.001 when compared with initial reading.

Blood glucose level of all rats before streptozotocin administration was within the normal range. Streptozotocin administration led to a significant increase (p < 0.001) in blood glucose level of diabetic rats compared to control rats. Treatment with pyridoxine alone and in combination with *Aegle marmelose *and insulin in diabetic rats was able to significantly reduce (p < 0.001) the increased blood glucose level to near the control value compared to diabetic group (Figure-1).

### Serotonin and Its Metabolites Content in Hippocampus of control and experimental rats

There was a significant decrease (p < 0.001) in 5-HT content in hippocampus of diabetic rats compared to control rats. The decreased 5-HT content was significantly reversed the D+P (p < 0.01), D+I (p < 0.01), DIP (p < 0.001), D+A (p < 0.01) and DAP (p < 0.001) to near control in diabetic rats treated with pyridoxine alone and in combination with insulin and *Aegle marmelose *leaf extract. The 5-HIAA in the hippocampus was significantly increased (p < 0.001) in diabetic rats compared to control. The increased 5-HIAA content was significantly reversed in D+P (p < 0.01), D+I (p < 0.01), DIP (p < 0.001), D+A (p < 0.01) and DAP (p < 0.001) to near control in diabetic rats treated with pyridoxine alone and in combination with insulin and *Aegle marmelose *leaf extract (Table-1).

**Table 1:** Serotonin and metabolites in the hippocampus of control and experimental rats

Experimental Groups	5-HT (nmoles/g wet wt. of tissue)	5HIAA (nmoles/g wet wt. of tissue)	5-HIAA/ 5-HT
**Control**	1.56 ± 0.27	1.94 ± 0.22	1.24 ± 0.23
**Diabetic**	0.89 ± 0.29 ^a^	2.93 ± 0.31 ^a^	3.29 ± 0.28 ^a^
**Diabetic+Insulin**	1.07 ± 0.19 ^a, b^	2.29 ± 0.20 ^a, b^	2.14 ± 0.20 ^a^
**Diabetic+Pyridoxine**	0.98 ± 0.33 ^a, b^	2.91 ± 0.36 ^a^	2.96 ± 0.33 ^a^
**Diabetic+Insulin+Pyridoxine**	1.45 ± 0.35 ^c^	1.53 ± 0.29 ^c^	1.05 ± 0.31 ^c^
**Diabetic+*A. marmelose***	1.10 ± 0.23 ^a, b^	2.73 ± 0.24 ^a, b^	2.48 ± 0.21 ^a^
**Diabetic+*A. marmelose*+Pyridoxine**	1.59 ± 0.22 ^c^	1.66 ± 0.22 ^c^	1.04 ± 0.21 ^c^

Values are mean ± S.E.M of 4-6 separate experiments. Each group consists of 6-8 rats.

^a ^*p *< 0.001 when compared to control;

^b ^p < 0.01, ^c ^*p *< 0.001 when compared to diabetic group.

### 5-HT and 5-HT_2A _receptor binding in the hippocampus of control and experimental rats

**Figure 2:**
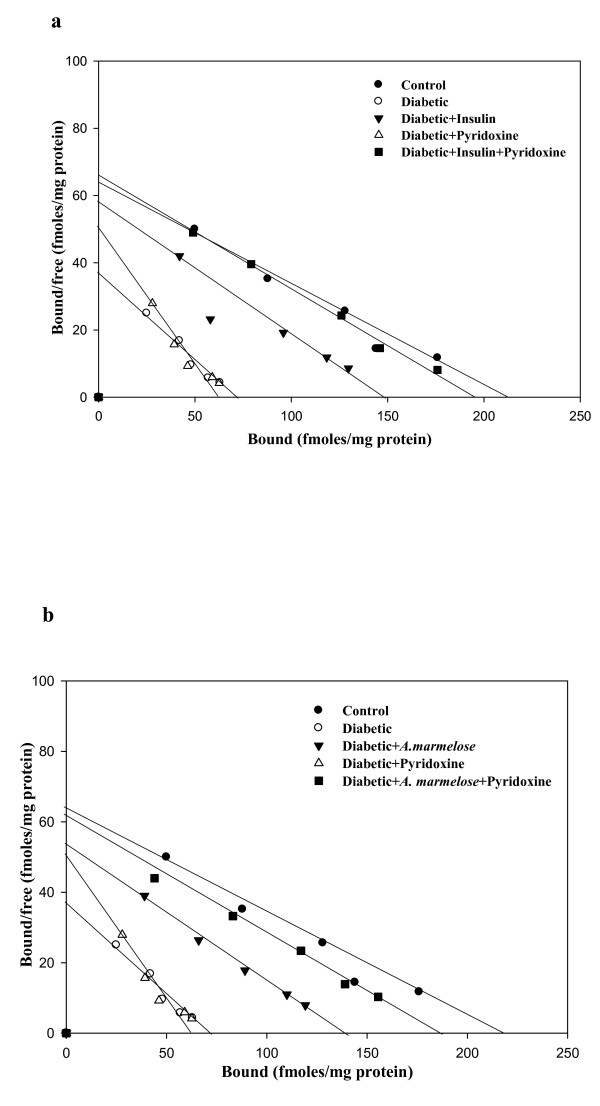
**a, b Representative graph showing Scatchard analysis of [^3^H] 5-HT binding against 5-HT in the hippocampus of control and experimental rats**. B_max _-- Maximal Binding (fmol/mg protein), K_d _-- dissociation constant (nM). Values are Mean ± S.E.M. of 4-6 separate experiments. Each group consists of 6-8 rats. ^a ^*p *< 0.001, ^b ^*p *< 0.05 when compared to control group; ^c ^*p *< 0.001 when compared to diabetic group. Incubation was done with 1.0 nM-30 nM at 37 °C of [^3^H] 5-HT in a total incubation volume of 250 μl. 10 μM unlabelled 5-HT was used to determine the nonspecific binding. The reaction was stopped by rapid filtration through GF/B filters using ice cold Washing Buffer pH 8.5. Bound radioactivity was counted with cocktail-T in a Wallac 1409 liquid scintillation counter.

Scatchard analysis using [^3^H] 5-HT binding against 5-HT showed that the B_max _decreased significantly (p < 0.001) in the hippocampus of diabetic rats with significant increase (p < 0.001) in the affinity. Treatment with pyridoxine alone and in combination with *Aegle marmelose *and insulin in diabetic rats reversed the B_max _and K_d _to near control compared to diabetic group (Table-2, Figure-2a, b).

**Table 2:** [^3^H] 5-Hydroxytryptamine binding parameters in the hippocampus of control and experimental rats

Experimental Groups	**B**_**max**_ (fmoles/mg protein)	**K**_**d **_**(nM)**
**Control**	212.5 ± 2.11	3.22 ± 0.54
**Diabetic**	72.4 ± 3.21 ^a^	1.94 ± 0.41 ^a^
**Diabetic + Insulin**	62.8 ± 2.06 ^a^	1.40 ± 0.29 ^a^
**Diabetic + Pyridoxine**	148.4 ± 2.33 ^a, c^	2.60 ± 0.49 ^b^
**Diabetic + Insulin+ Pyridoxine**	196.0 ± 1.43 ^c^	3.20 ± 0.17 ^c^
**Diabetic+*A. marmelose***	140.1 ± 4.33 ^a, c^	2.57 ± 1.42 ^b^
**Diabetic+ *A. marmelose *+Pyridoxine**	186.4 ± 2.42 ^c^	3.05 ± 1.31 ^c^

Values are mean ± S.E.M of 6-8 separate experiments. Each group consists of 6-8 rats.

^a ^*p *< 0.001, ^b ^*p *< 0.05 when compared to control group; ^c ^*p *< 0.001 when compared to diabetic group.

**Figure 3:**
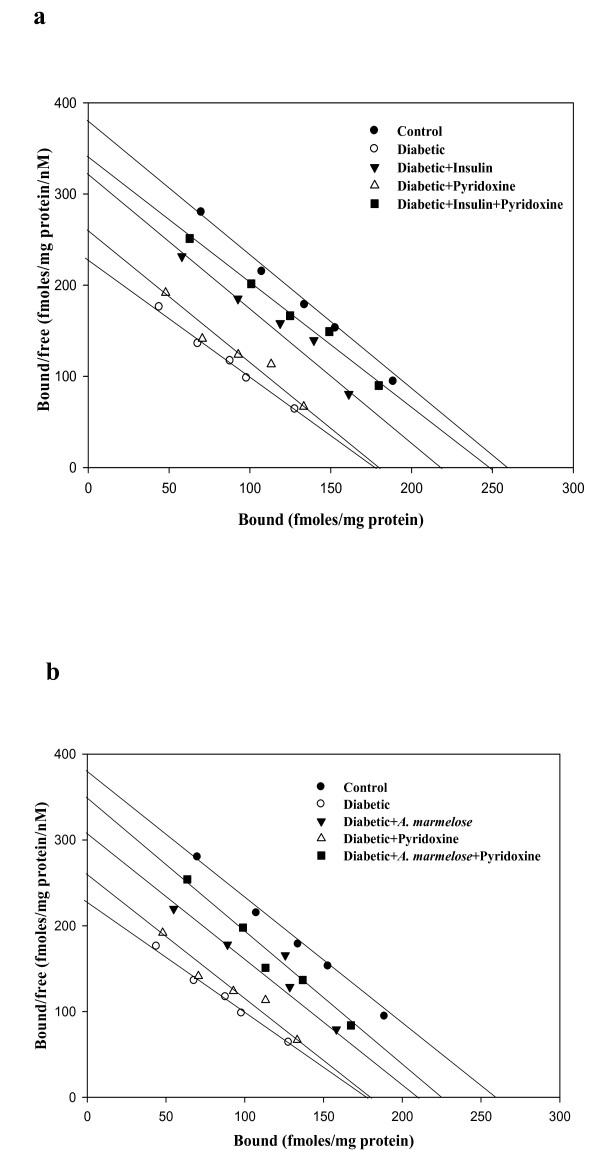
**a, b Representative graph showing Scatchard analysis of [^3^H] Ketanserin binding against ketanserin in the hippocampus of control and experimental rats**. B_max _-- Maximal Binding (fmol/mg protein), K_d _-- dissociation constant (nM). Values are mean ± S.E.M of 4-6 separate experiments. Each group consists of 6-8 rats. ^a ^*p *< 0.05, ^b ^p < 0.001 when compared to control; ^c ^*p *< 0.05, ^d ^p < 0.001 when compared to diabetic group. Incubation was done with 0.1 nM-2.5 nM at 37 °C of [^3^H] Ketanserin in a total incubation volume of 250 μl. 10 μM unlabelled ketanserin was used to determine the nonspecific binding. The reaction was stopped by rapid filtration through GF/B filters using ice cold Washing Buffer pH 7.6. Bound radioactivity was counted with cocktail-T in a Wallac 1409 liquid scintillation counter.

Scatchard analysis using [^3^H] Ketanserin binding against ketanserin showed that the B_max _decreased significantly (p < 0.001) in the hippocampus of diabetic rats with significant increase (p < 0.001) in the affinity. Treatment groups reversed the B_max _of D+I (p < 0.001), DIP (p < 0.001), D+A (p < 0.001) and DAP (p < 0.001) to near control compared to diabetic group (Table-3, Figure-3a, b).

**Table 3:** [^3^H] Ketanserin binding parameters in the hippocampus of control and experimental rats

Experimental Groups	B_max_ (fmoles/mg protein)	**K**_**d **_**(nM)**
**Control**	260.5 ± 0.35	0.68 ± 0.08
**Diabetic**	176.2 ± 0.19 ^b^	0.77 ± 0.17 ^a^
**Diabetic + Insulin**	218.1 ± 0.32 ^d^	0.67 ± 0.09 ^c^
**Diabetic + Pyridoxine**	180.6 ± 0.27 ^b^	0.70 ± 0.06 ^c^
**Diabetic + Insulin+ Pyridoxine**	244.0 ± 0.26 ^d^	0.68 ± 0.11 ^c^
**Diabetic+*A. marmelose***	209.3 ± 0.22 ^d^	0.68 ± 0.11 ^c^
**Diabetic+ *A. marmelose *+Pyridoxine**	228.2 ± 0.29 ^d^	0.67 ± 0.07 ^c^

Values are mean ± S.E.M of 6-8 separate experiments. Each group consists of 6-8 rats.

^a ^*p *< 0.05, ^b ^p < 0.001 when compared to control;

^c ^*p *< 0.05, ^d ^p < 0.001 when compared to diabetic group.

### Real Time-PCR analysis of 5-HT_2A, _5-HTT and INSR receptor expression in the hippocampus of control and experimental rats

**Figure 4:**
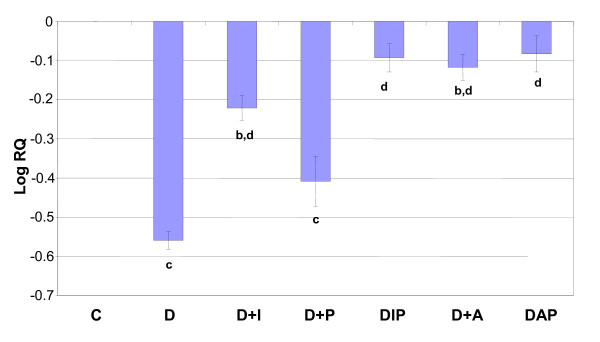
**Representative graph showing Real Time amplification of 5-HT_2A _mRNA from the hippocampus of control and experimental rats**. are mean ± S.E.M of 4-6 rats in each group. Each group consists of 6-8 rats. ^a ^*p *< 0.001 when compared to control group, ^b ^*p *< 0.001 when compared to diabetic group. The relative ratios of mRNA levels were calculated using the ΔΔCT method normalized with β-actin CT-value as the internal control and Control CT-value as the calibrator.

**Figure 5:**
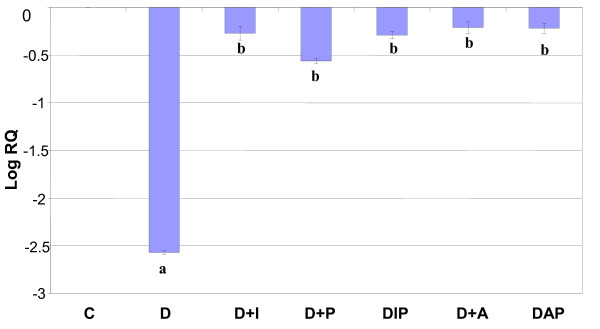
**Representative graph showing Real Time amplification of 5-HTT mRNA from the hippocampus of Control and experimental rats**. are mean ± S.E.M of 4-6 rats in each group. Each group consists of 6-8 rats. ^a ^*p *< 0.05, ^b ^*p *< 0.001 when compared to control group, ^c^*p *< 0.001 when compared to diabetic group. The relative ratios of mRNA levels were calculated using the ΔΔCT method normalized with β-actin CT-value as the internal control and Control CT-value as the calibrator.

Real Time-PCR analysis showed that the 5-HT_2A _and 5-HTT mRNA showed a significant down regulation (p < 0.001) in diabetic rats when compared to control and it was (p < 0.001) reversed to near control level on treatment with pyridoxine alone and in combination therapy with *Aegle marmelose *and insulin in diabetic rats (Figure-4, 5).

**Figure 6:**
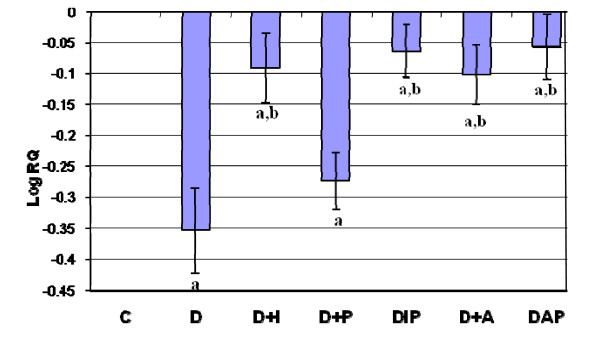
**Representative graph showing Real Time amplification of INSR mRNA from the hippocampus of Control and experimental rats**. are mean ± S.E.M of 4-6 rats in each group. Each group consists of 6-8 rats. ^a ^*p *< 0.05, ^b ^*p *< 0.001 when compared to control group, ^c^*p *< 0.001 when compared to diabetic group. The relative ratios of mRNA levels were calculated using the ΔΔCT method normalized with β-actin CT-value as the internal control and Control CT-value as the calibrator.

Real Time-PCR analysis showed that the INSR mRNA showed a significant down regulation (p < 0.001) in diabetic rats when compared to control and it was (p < 0.001) reversed to near control level on treatment with pyridoxine alone and in combination therapy with *Aegle marmelose *and insulin in diabetic rats (Figure-6).

### Elevated plus maze test in the control and experimental rats

(i) Behavioural response in streptozotocin induced diabetic Rats: Effect of insulin and pyridoxine treatment on open and closed arm entry in elevated plus- maze test

**Figure 7:**
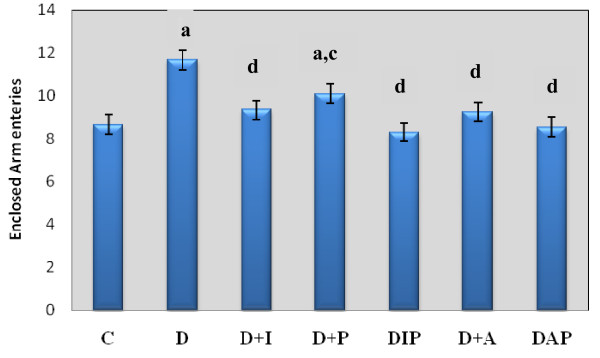
**Representative graph showing behavioural response in streptozotocin induced diabetic Rats: Effects of insulin and pyridoxine treatment and Closed Arm Entry attempts (Counts/5 minutes) in Elevated plus- maze test by of control and experimental rats**. Values are mean ± S.E.M of 4-6 separate experiments. Each group consists of 6-8 rats. ^a ^*p *< 0.001 when compared to control group; ^c ^*p *< 0.01, ^d ^*p *< 0.001 when compared to diabetic group.

The experimental groups showed a significant increase in the attempt taken for open arm entry- D (p < 0.001) compared to C. D+I (p < 0.001), D+P (p < 0.01), DIP (p < 0.001), D+A(p < 0.001) and DAP (p < 0.001) treated groups showed the open arm entry to near control (Figure-7).

**Figure 8:**
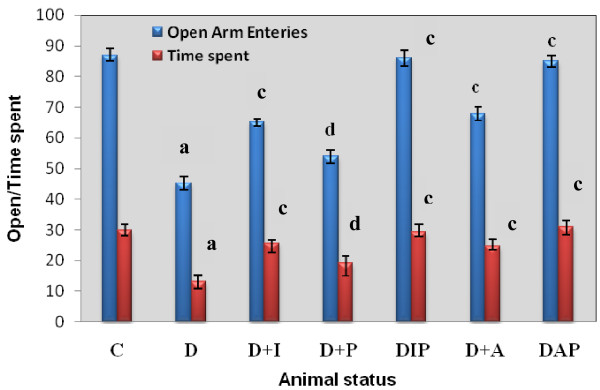
**Representative graph showing behavioural response in streptozotocin induced diabetic Rats: Effects of insulin and pyridoxine treatment in Time Spent in Open Arm Entry attempts (Counts/5 minutes) by control and experimental rats in Elevated plus- maze test by of control and experimental rats**. Values are mean ± S.E.M of 4-6 separate experiments. Each group consists of 6-8 rats. ^a ^*p *< 0.001 when compared to control group; ^c ^*p *< 0.01, ^d ^*p *< 0.001 when compared to diabetic group.

There was a significant increase (p < 0.001) in the number of entries made into closed arm by D compared to C. D+I (p < 0.001), D+P (p < 0.01), DIP (p < 0.001), D+A (p < 0.001) and DAP (p < 0.001) treated groups showed the open arm entry to near control (Figure-7, 8).

(ii) Behavioural response in streptozotocin induced diabetic Rats: Effects insulin and pyridoxine treatment on time spent in open and closed arms in Elevated plus-maze test

There was a significant decrease in time spent in open arm by D (p < 0.001) compared to C (Figure-7). Time spent in closed arm showed a significant increase in D (p < 0.001) when compared to C. D+I (p < 0.001), D+P (p < 0.01), DIP (p < 0.001), D+A (p < 0.001) and DAP (p < 0.001) treated groups showed the time spent in open and closed arms near to control (Figure-8).

### 5-HT_2A _and 5-HTT antibody staining in control and experimental groups of rats

**Figure 9:**
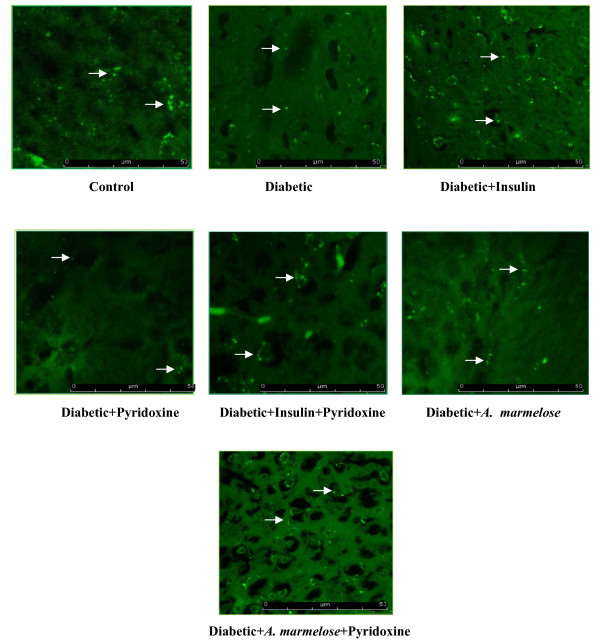
**Confocal image of 5-HT_2A _receptors in the hippocampus of control and Experimental rats using immunofluorescent 5-HT_2A _receptor specific primary antibody and FITC as secondary antibody**. The pixel intensity of The pixel intensity of control - 384132 ± 1454, diabetic - 133475 ± 1431 ^a^, diabetic+Insulin - 229123 ± 1453 ^a, c^, diabetic+pyridoxine - 151012 ± 2662 ^a, b^, diabetic+insulin+pyridoxine - 398791 ± 2105 ^c^, diabetic+*Aegle marmelose - *222921 ± 1097^a, c ^and diabetic+*Aegle marmelose+*pyridoxine - 314997 ± 1084 ^c^. Values are mean ± S.E.M of 4-6 rats in each group. Each group consists of 6-8 rats. ^a ^*p *< 0.001 when compared to control group; ^b ^*p *< 0.05, ^c ^*p *< 0.001 when compared to diabetic group. There was significant reversal of 5-HT_2A _receptor to near control level on treatment with pyridoxine alone and in combination therapy with insulin and *Aegle marmelose *compared to diabetic rats. Arrow in white shows 5-HT_2A _receptors.

The 5-HT_2A _receptor antibody staining in the hippocampus showed significant decrease (p < 0.001) in the 5-HT_2A _receptor in diabetic rats compared to control. There was significant reversal of 5-HT_2A _receptor to near control level in D+I (p < 0.001), D+P (p < 0.05), DIP (p < 0.001), D+A (p < 0.001) and DAP (p < 0.001) of 5-HT_2A _receptors on treatment with pyridoxine alone and in combination therapy with insulin and *Aegle marmelose *compared to diabetic rats (Figure-9).

**Figure 10:**
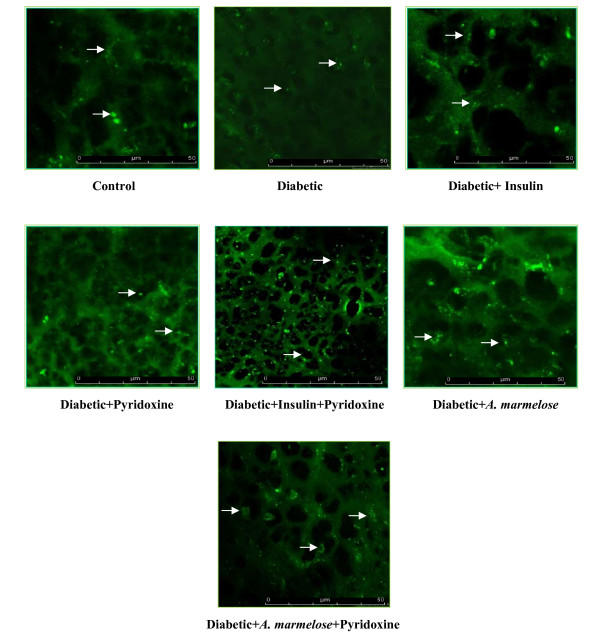
**Confocal image of 5-HTT receptors in the hippocampus of control and Experimental rats using immunofluorescent 5-HTT receptor specific primary antibody and FITC as secondary antibody**. The pixel intensity of control - 4235653 ± 1960, diabetic - 2833408 ± 1978 ^a^, diabetic+Insulin - 3964668 ± 1670^a, b^, diabetic+pyridoxine - 2897587 ± 3426^a^, diabetic+insulin+pyridoxine - 4121017 ± 2723^b^, diabetic+*Aegle marmelose - *3866844 ± 1534^a, b ^and diabetic+*Aegle marmelose+*pyridoxine - 4098789 ± 2086 ^b^. Values are mean ± S.E.M of 4-6 rats in each group. Each group consists of 6-8 rats. ^a ^*p *< 0.001 when compared to control group; ^b ^*p *< 0.001 when compared to diabetic group. There was significant reversal of 5-HTT receptor to near control level on treatment with pyridoxine alone and in combination therapy with insulin and *Aegle marmelose *compared to diabetic rats. Arrow in white shows 5-HTT.

The 5-HTT antibody staining in the hippocampus showed significant decrease (p < 0.001) in the 5-HTT in diabetic rats compared to control. There was a significant reversal to near control level in expression of D+I (p < 0.001), DIP (p < 0.001), D+A (p < 0.001) and DAP (p < 0.001) of 5-HTT on treatment with insulin and *Aegle marmelose *alone and in combination therapy with insulin and *Aegle marmelose *compared to diabetic rat (Figure-10).

## Discussion

Maintenance of euglycemia over a lifetime of diabetes cannot be accomplished safely with currently available treatment methods [26]. The effect of hyperglycemic episodes is visible in brain regions associated with memory, especially the hippocampus [27]. Increased blood glucose level observed during diabetes is similar with previous reports as a result of the marked destruction of insulin secreting pancreatic β-cells by streptozotocin [28]. Treatment normalised the increased blood glucose level to control. A decrease in the rate of 5-HT synthesis and changes in 5-HT neurotransmission have demonstrated to reduce 5-HT concentrations [29]. In the brain, serotonergic fibres acts on specific receptors to modulate the activity on autonomic pathways and affects energy expenditure regulated by 5-HT receptors. Serotonergic pathways also directly affect glucose homeostasis through regulation of autonomic efferents and action on peripheral tissues [30].

5-HT has both depolarising and hyperpolarizing effects in the hippocampus, *via *its different receptors. Activation of 5-HT_2A _receptors found in the hippocampus has been suggested to induce depolarization in the dentate gyrus [31]. 5-HT_2A _receptor has been found to enhance Long term potentiation in the hippocampus [32]. The changes in brain 5-HT synthesis rate in diabetic rats are related to the various behavioural and psychological changes. The psychological changes observed in diabetes appear to persist even when the diabetic state is well-controlled with insulin administration [33].

Previous reports showed a decrease in 5-HT in brain regions during diabetes [29]. 5-HIAA/5-HT turnover ratio showed an increase in diabetes. In hippocampus, inactive decarboxylation reaction due to lack of pyridoxal phosphate decreased the conversion to 5-HT. Treatment of rats with moderate doses of pyridoxine results in an increment in brain 5-HT indicating that the tissue 5-HTP decarboxylation responds to the pyridoxine status of the animal [34]. Present study indicates a decreased 5-HT and 5-HT_2A _receptor binding with increase in affinity in hippocampus of diabetic rats. This decrease in the sympathetic activity thereby decreases the circulating 5-HT level. Treatment of pyridoxine along with *Aegle marmelose *and insulin, resulted in restoring the synthesis of 5-HT in hippocampus of diabetic rats. 5-HT levels reflect the intrasynaptic release indicated by the response of the B_max _of 5-HT receptor binding to its ligand. The results indicate that the pyridoxal phosphate content in hippocampus regulates the extent of decarboxylation of the 5-HTP, the precursor of 5-HT. Treatment of diabetic rats with pyridoxine reflected the synthesis and secretion into the synaptic cleft of the neurotransmitter 5-HT [35,36]. 5-HT synthesis is increased, possibly as a result of desensitization of receptors [37] and thereby modifying synthesis and release of 5-HT.

5-HTT regulates the entire serotonergic system and its receptors *via *modulation of its expression and function. In brain, 5-HTT is situated both in presynaptic membranes of nerve terminals in proximity to serotonin-containing cell bodies [38]. 5-HTT mediates rapid removal and recycling of released 5-HT following neuronal stimulation. Thus, it has a critical role in the homeostatic regulation of the signals reaching 5-HT receptors. 5-HTT is important in emotion regulation and social behaviour, drawing from an interdisciplinary perspective of behavioural genetics and cognitive neuroscience. Integration of these findings suggest that the 5-HTT gene has an impact on behaviour and have a role in social cognition [39]. 5-HT is packaged into vesicles for synaptic exocytosis. Extracellular 5-HT signals through 5-HT_2A _receptors. Synaptic 5-HT signaling are motivated by uptake of 5-HT_2A _from the synapse by 5-HTT.

Recent evidence suggests that a dysfunction of the neuronal insulin receptor signalling cascade, with the subsequent abnormalities in glucose/energy metabolism, affect amyloid precursor protein metabolism and cause insulin dysfunction [40]. In this study the altered expression of insulin receptor expression in the hippocampus of diabetic rats was reversed to near control by treatment with insulin and *Aegle marmelose *alone and in combination with pyridoxine. The distribution of insulin receptors in the brain and the presence of insulin-dependent glucose transporters suggest that brain insulin participate in several cognitive functions, including learning and memory [41]. In animal models of diabetes, impairments of spatial learning occur in association with distinct changes in hippocampal synaptic plasticity due to defects in insulin action in the brain [42]. Treatment with insulin therefore not only corrects hyperglycaemia, but also directly affects the brain. One problem is that exogenous insulin injection reduces blood glucose and lead to hypoglycaemia that is associated with impaired memory [43]. Cognitive impairments associated with diabetes caused by inadequate insulin/insulin receptor functions have also been documented [44]. The role of insulin as a regulator for cell proliferation has already been established [45]. It was observed from the earlier studies that administration of pyridoxine along with insulin serves as a control measure for diabetes, regulating GDH activity and glucose level [14]. The reversal of hyperglycaemic condition in DIP treatment group is due to the effect of pyridoxine and insulin on pancreatic β cells. Treatment with pyridoxine to diabetic rats caused a reversal in the B_max _of 5-HT_2A _receptors to near control level. Also, it is evident that pyridoxine along with insulin and *Aegle marmelose *leaf extract has neuroprotective action mediated through the 5-HTT at the transcription level.

*Aegle marmelose *was comparable to insulin in reversing blood glucose to normal levels. Anandharajan et al. [46] reported that *Aegle marmelose *activate glucose transport in PI3 kinase-dependent fashion. Scopoletin (7-hydroxy-6-methoxy coumarin) isolated from leaves of *Aegle marmelose *was evaluated for its potential to regulate hyperglycemia in rats [47]. Alkaloidal-amide Aegeline isolated from leaves of *Aegle marmelose *is found to have anti-hyperglycemic activity by lowering the blood glucose [46]. The leaf extract treated animals appeared healthier and were less prone to the frequent hypoglycaemic condition observed in their insulin treated counter parts. Previous studies by Sharma et al. [48] the plant extract enhance glucose utilization since it significantly decreased the blood glucose level. This fact attributed to potentiating of insulin effect of plasma by increasing the pancreatic secretion of insulin from existing β-cell or its release from bound insulin intercede by increase in the insulin receptor. Treatment with pyridoxine alone and in combination with insulin and *Aegle marmelose *to diabetic rats caused a reversal in the B_max _of 5-HT, 5-HT_2A _receptors and gene expression to near control level. Also, it is evident that *Aegle marmelose *has a role in controlling INSR function. This study demonstrates the involvement of 5-HT_2A _receptor which has modulating effect on the diabetes stress. Administration of pyridoxine alone and in combination with *Aegle marmelose *and insulin significantly decreased the diabetic associated stress. Hence the treatment has pharmacological and neurobiological basis.

The change in brain 5-HT synthesis rate in diabetic animals is related to the various behavioural and psychological changes [49]. Anxiety is a neurological problem associated with diabetes mellitus. Muneoka and colleagues [50] revealed the correlation between diabetic anxiety and serotonergic systems. There is a decrease in the serotonergic response to stressful stimuli and the dysfunction of stress-elicited 5-HT release caused the increased expression of fear-related behaviour in diabetic rats [51]. Investigation on elevated plus maze and spontaneous alternation in behaviour paradigm as a measure of anxiety. Our findings support impaired hippocampal plasticity and cognition induced by diabetes. Diabetic rats showed an increased percentage attempt made towards open arm entry and the animal also remained for longer period in closed arms of elevated plus-maze maze thereby causing hypo locomotion in diabetic rats. The treatment reversed the behavioural deficit in diabetic rats to near control. The possible anxiolytic effect is related to its effect on serotonergic transmission [52]. Thus perturbations of the 5-HT receptor system directly modulate stress susceptibility rendering them anxiogenic as well as depressive profile. A decrease in general exploratory activity in an open arena after restraint stress has been previously described [37]. In the elevated plus-maze studies found a decrease in the percentage of open arm entries and/or time spent in them [6,29]. This study demonstrates the involvement of 5-HT receptor which has modulating effect on the diabetes and associated motor defects. Cools et al. [53] suggest that serotonergic receptor function resolve the essential inconsistency in hippocampus associated with depression. Administration of pyridoxine and insulin significantly increased the percentage of open arm entries and the number of total entries. Hence the treatment has pharmacological and neurobiological bases of anxiety.

The prevalence of diabetes among depressed and anxious patients is due to high sensitivity of diabetics to the adverse effects of stress, etiology and course of the disease. Serotonergic system and insulin function play key roles in the regulation of stress-related behaviours. The result of this study has demonstrated the effect of insulin, *Aegle marmelose *leaf extract alone and in combination with pyridoxine has marked to normalize 5-HT, 5-HT_2A _receptor, gene expression studies along with the Elevated plus test implicate a role in reducing the stress associated with diabetic rats. Thus it is suggested that pyridoxine treated alone and in combination with insulin and *Aegle marmelose *have a functional role through serotonergic receptors regulation in the hippocampus. This represents a possibility for the better management of diabetic mediated neurological complications.

## Abbreviations

INSR: Insulin receptor; 5-HT: Serotonin; 5-HIAA: 5-hydroxy indole acetic acid; 5-HTT: 5-HT Transporter; EDTA: Ethylene Diamine Tetra Acetic Acid.

## Competing interests

The authors declare that they have no competing interests.

## Authors' contributions

PMA and CSP designed research. PMA, KPK, JM, AM and SJ carried out the experiments and drafted manuscript. All authors read and approved the final manuscript.
